# The effect of biological rhythms and personality traits on the incidence of unsafe behaviors among bus drivers in Shiraz, Iran

**DOI:** 10.5249/jivr.v10i1.895

**Published:** 2018-01

**Authors:** Fatemeh Kamari Ghanavati, Mehdi Jahangiri, Maryam Khalifeh, Sareh Keshavarzi, Mahnaz Shakerian

**Affiliations:** ^*a*^Department of Occupational Health Engineering, School of Public Health, Shiraz University of Medical Sciences, Shiraz, Iran.; ^*b*^Department of Epidemiology, School of Public Health, Shiraz University of Medical Sciences, Shiraz, Iran.

**Keywords:** Biological, Rhythms, Personality Traits, Unsafe Behavior, Bus Drivers

## Abstract

**Background:**

Unsafe behaviors are main causes of accidents mostly influenced by personal characteristics, social environment and also individual biorhythm cycles. This study was carried out to find out whether personality traits and biorhythm could affect the incidence of unsafe behaviors among city bus drivers.

**Methods:**

This cross-sectional study was conducted on 224 bus drivers in Shiraz, Iran, 2014. The data were collected using NEO personality traits questionnaire. Also, a self-constructed checklist was used to investigate the drivers' unsafe behaviors. Information on drivers' biorhythm was analyzed by Natural Biorhythm software version 3.2. The relationship between demographic characteristics, personality traits and biorhythm were examined by T-Test, One-way ANOVA, correlation coefficient and Chi square tests. Multiple linear regression analysis was used to investigate the factors influencing the incidence of unsafe behaviors.

**Results:**

28.6% of participants have experienced critical days in their biorhythm cycles. Also the mean percentage of unsafe behavior was 54.08 ± 11.91 among the subjects of the study. Significantly negative correlations were observed between each personality factor and the percentage of unsafe behaviors. Also, there was a significant relationship between percentage of derivers' unsafe behaviors and the general cycle of biorhythm (having at least one critical day in each of the cycles).

**Conclusions:**

Taking the measures including selection of low-risk traffic routes in the critical days and considering the personality traits at the time of employment could be effective in reducing the unsafe behaviors and accidents.

## Introduction

Iran has been ranked first in the world in terms of traffic accidents in proportion to her population and the number of vehicles. The annual number of people killed and injured by traffic accidents has been estimated at between 27,000 and 250,000, respectively.^1^ Based on previous studies four main factors are responsible for traffic accidents namely human, road, vehicle and environment.^2^ Although unsafe conditions and unsafe behaviors are two main causes of accidents,^3^ many vehicle accidents may result from either driving malfunction or unsafe behaviors. Therefore, the impacts due to unsafe behaviors seem to be more important.^4,5,6^ Driving is considered as a set of complex and dynamic actions (behaviors) which by itself is a process of controlling activities^7^ Driving behavior is a behavioral model through which a driver would choose how to drive.^8^ High risk driving behavior means the act of committing two or more violations that puts people or other vehicles at risk and requires defensive response from individuals or from other drivers.^9^ Normal or safe driving is considered as an activity with controlled movements where the driver receives driving-related information through different ways especially the visual route which entails making decisions in the mind and putting these decisions into practice.^9^ Therefore, the identification of individual-induced causes leading to unsafe behaviors seems very useful to control unsafe behaviors at work. Unsafe behaviors can be due to individual and social characteristics.^10^ On the other hand, each person has features that can contribute to certain behaviors in a regular and permanent manner.^11^ It seems that there is a significant relationship between personality dimensions and the extent of unsafe behaviors.^12^ Since the unsafe behaviors are largely influenced by personality traits, it may be applied as a predictive tool for unsafe behaviors.^12^

In some studies, individual biorhythm cycles are named as one of the factors affecting unsafe behaviors and occupational accidents. ^13-15^ Biorhythm can describe the energy levels and the performance capacities in physical, emotional and intellectual aspects. ^16^ Biorhythm theory was introduced in 1890 by two German physicians (Latman and Garriott) and was gradually expanded. ^15^ The word biorhythm is derived from the Greek word “bio” meaning life, and rhythmus meaning a systematic and deliberate motion. From the perspective of biorhythm theory, every human is affected by three internal cycles namely: 1) physical cycle (with a period of 23 days), 2) emotional cycle (with a period of 28 days) and 3) intellectual cycle (with a period of 33 days) and these effects continue from birth to death. ^17^ During the time that a cycle moves from the positive to the negative phase or vice versa, the ability to function in areas related to that cycle becomes very unstable. ^18^ During such days called critical days, humans become prone to making errors which likely followed by accidents.^19^

According to the facts mentioned above and considering the limited studies conducted in this field, this study was carried out to determine whether personality traits and biorhythm could affect the incidence of unsafe behaviors among city bus drivers. 

## Methods 

**Sampling and Data Collection**

This cross-sectional study was conducted on bus drivers in the city of Shiraz, Iran, 2014.

From a list of 1900 Personnel ID of Bus drivers employed in 12 bus terminals of Shiraz Bus Operating Company (SBOC), 262 drivers were selected by stratified random sampling. The inclusion criteria comprised of (1) being experienced as a bus driver at least for one year, and (2) not having a second job. From the participants, 38 drivers were set aside as they did not meet the inclusion criteria of the study, their questionnaires were not completed readably or they did not agree to complete the NEO questionnaire after their behavioral observation. All participants were of the male gender and their average age and work experience were 42.29 ± 9.27 and 17.97±8.05 years, respectively (Table 1).

**Table 1:** Demographic characteristics of the study population (n = 224).

Education	Under diploma	92(41.4%)
	Diploma	89 (39.7%)
	Associate diploma	36 (15.3%)
	B.Sc.	8 (3.6%)
Working hours per days	8	149 (66.5%)
	16	75 (33.5%)
Age	M (±SD)	42.29 (9.27)
	Min-Max	23-61
Work experience (yrs)	M (±SD)	17.97 (8.05)
	Min-Max	2-30

**Measures**

Data collection was carried out with the use of a Bus Driver Behavior (BDB) checklist, NEO Personality Inventory (Short Form) and the Natural Biorhythm software.

**Bus Driver Behavior Checklist (BDB)**

Required data related to the behaviors of bus drivers were collected using a “Bus Driver Behavior (BDB)” checklist constructed based on regulations of SBOC. The checklist covered twenty items, from which, four items (No. 17 to 20) were scored.

The observers marked the behaviors of drivers in the Yes or No column. The percentage of unsafe behaviors was calculated by the sum of unsafe behaviors number (Number of "No" replies plus number of "Yes" replies for reverse scoring items) by total number of items multiplied by 100. Obtaining a higher percentage of unsafe behavior means a more unsafe driving situation. The items of the checklist were checked by three experienced personnel working in the SBOC in terms of relevance, clarity and simplicity stand point and all required corrections were made on the related items to be considered. Moreover, the reliability of the checklist, was measured with the Cronbach’s alpha test and the result revealed an acceptable internal consistency for the checklist (a=0.87). The inter-rater reliability of checklist was also checked by Intra-class Correlation Coefficient (ICC). For this purpose, five bus drivers were rated by five (5) observers and the results showed an acceptable inter-rater reliability (ICC=0.95).

**NEO Personality Inventory**

In this study, the Persian version of the standardized questionnaire NEO-FFI (NEO -Five Factor Inventory) was used in a short form to evaluate the personality traits among bus drivers.^20^ This questionnaire assesses five dimensions of personality namely: Neuroticism (the tendency to experience negative emotions and psychological distress in response to stressors), Extraversion (the degree of sociability, positive emotionality, and general activity), Openness (the levels of curiosity, independent judgment and conservativeness), Agreeableness (altruistic, sympathetic and cooperative tendencies) and Conscientiousness (one’s level of self-control in planning and organization). Each dimension consisted of twelve (12) items (totalling 60 items). Each item received a score of 0 to 4 according to a 5-choice Likert scale, including strongly disagree, disagree, no idea, agree and completely agree. ^21^ Higher score means higher personality traits in each dimension. In Iran, Haghshenas has confirmed the reliability of this test through its implementation on a sample of 502 people in Shiraz, using both the test-retest technique and Cronbach's alpha.^22^ Moreover, Garousi reported an internal consistency of 0.86, 0.73, 0.77, 0.68 and 0.87, respectively for Neuroticism, Extraversion, Openness, Agreeableness and Conscientiousness dimensions, among Iranian students.^23^

**Natural Biorhythm software**

The required information related to the biorhythm of the study population was analyzed using the Natural Biorhythm software version 3.2. For this purpose, the exact date of birth (AD) and the date of behavior observation for each participant were entered in the software to determine the physical, intellectual and emotional cycles of individuals as well as the critical days of each cycle. Critical days means the time that a cycle (physical, emotional and intellectual) moves from the positive to negative phase or vice versa. 

Those participants with at least one critical day in one of their biorhythm cycles were classified as “individual with critical days". Then, the relationship between the biorhythm state of individuals and the incidence of observed unsafe behaviors at the date of observing behaviors was investigated.

**Procedure**

The study protocol was approved by the Shiraz University of Medical Sciences ethics committee and SBOC was informed about the objectives of the study. Sampling the behavior of drivers was conducted by five male trained observers. For this purpose, the observers were asked to locate the nearest possible point to the driver (mostly seated in the first row of the bus seats), observe the drivers’ behavior directly and complete the BBC. Each observation was conducted continually for 30 minutes at the morning working shift. The company agreed to receive the results of drivers’ behavioral observations anonymously. Therefore, the sampling of drivers’ behavior did not affect their job security. The drivers under the study were not aware that they were being observed. After behavior observation and before completing the NEO questionnaire, drivers were informed about the objectives of the study and were asked to provide a written consent. In case of unwillingness to complete the NEO inventory, their completed BDB were put aside.

**Statistical analysis**

Statistical analysis was performed using SPSS version 19. Descriptive analysis was used to describe the variables including mean and standard deviation.

The independent t-test was used to compare quantitative variables with the two qualitative groups (individuals with at least one critical day in their biorhythm cycle with individuals who have not). The Pearson correlation was used to examine the relationships between quantitative variables. In order to compare the mean percent of unsafe behaviors in different levels of drivers' education, ANOVA test was applied. Moreover, the amount of independency or dependency between the two qualitative variables was investigated using chi-square test. Also, linear regression analysis was applied in order to assess the factors affecting unsafe behaviors. In all tests, the significance level was set at 0.05.

## Results

Table 2 shows the inter correlations between all study variables. The correlation between the personality trait score, in dimensions of neurosis (r=0.141, p=0.035), agreeableness (r= -0.281, p= 0.0001), extraversion (r= -0.310, p= 0.0001), conscientiousness (r= -0.239, p= 0.0001) and openness (r= -0.266, p= 0.0001) were negative and significant with the percentage of unsafe behaviors. The relationship between the mean percentage of unsafe behaviors and study variables are presented in Table 3. Table 4 shows the mean, standard deviation and a range for dimensions of personality traits Index (Table 4). In this study, there was a significant relationship between the percentage of unsafe behaviors (p= 0.0001), emotional cycle and general cycle of biorhythm (having at least one critical day in each of the cycles) (p= 0.001 and p= 0.0001, respectively), meaning a higher value of the average of unsafe behavior in the critical days (Table 5). The mean percentage of unsafe behavior in the subjects of the current study was 54.08 ± 11.91. In this study, 28.6% (n= 64) of study participants had critical days in their biorhythm cycles. Among the unsafe behaviors committed by drivers, the highest frequency belonged to the “Failing to wait for all passengers to take their seats before driving off” (93.3%), “Failure to fasten seat-belt” (92.4%) and “Forgetting to look in the appropriate mirrors before driving” (86.6%) (Table 6).

**Table 2:** Inter-correlations between all study variables.

Variables	Pearson correlation coefficients									
	1	2	3	4	5	6	7	8	9	10
Neuroticism	1	0.09	0.312**	0.268**	0.168*	0.141*	-0.221**	0.022	0.118	0.022
Extraversion		1	0.722**	0.763**	0.787**	-0.310**	0.210**	0.168*	0.078	0.168*
Openness			1	0.714**	0.696**	-0.266**	0.121	0.031	0.44	0.031
Agreeableness				1	0.810**	-0.281**	0.164*	0.011	0.059	0.011
Conscientiousness					1	-0.239**	0.197**	0.059	0.102	0.059
Unsafe behaviors						1	0.033	-0.163*	-0.106	-0.001
Age							1	0.111	0.078	0.948**
Income								1	0.776	0.112
Working hours									1	0.068
Work experience										1

٭٭ Correlation is significant at the 0.01 level (2- tailed) ٭ Correlation is significant at the 0.05 level (2- tailed)

**Table 3:** Relationship between the mean percentage of unsafe behaviors and study variables (n=224).

Variables	Mean percent of unsafe behaviors (SD)	p-value
Education		
Under diploma	53.42 (11.53)	
diploma	54.92 (12.89)	0.631*
Associate diploma	53.49 (12.67)	
B.Sc.	51.52 (14.57)	
At least one critical day in each cycles		
Yes	58.72 (11.26)	0.001†
No	52.03 (13.16)	
Biorhythm Physical cycles		
Yes	55.82 (14.53)	0.13†
No	57.27 (9.84)	
Biorhythm emotional cycles		
Yes	63.13 (13.17)	0.001†
No	53.7 (11.83)	
Biorhythm Intellectual cycles		
Yes	57.29 (12.33)	0.187†
No	53.7 (11.83)	

* One-Way ANOVA Test, † Independent Samples T-Test

**Table 4:** Mean, standard deviation and range for dimensions of personality traits Index (n=224).

Personality traits dimensions	Mean	SD	Min	Max
Neuroticism	24.80	5.36	14	44
Extraversion	35.78	5.17	24	48
Openness	33.53	3.97	26	47
Agreeableness	38.46	3.93	31	48
Conscientiousness	40.35	4.51	25	48

**Table 5:** The relationship between the percentage of unsafe behaviors and biorhythm cycles in the subjects of the study (n = 224).

Biorhythm cycles	Index		
	T	CI95	p-value
Physical cycle	-1.35	-9.02_1.84	0.187
Emotional cycle	-3.60	-15.85_-4.37	0.001
Intellectual cycle	-1.56	-8.17_1.10	0.13
At least one critical day in each cycles	-4.11	-10.36_-3.72	0.0001

**Table 6:** Results of bus drivers’ behaviors analysis in the study subjects.

Items	N (%)	
	Yes	No
1. Full stop at the bus station when boarding and alighting the passengers	192 (85.7)	32 (14.3)
2. Completely stop the bus and then open the door	84 (37.5)	140 (62.5)
3. Ensuring about closing the door before starting to move	91 (40.6)	133 (59.4)
4. Check the mirror when boarding and alighting the passengers at stations	98 (43.8)	126 (56.3)
5. Carefully check the mirrors before moving	30 (13.4)	194 (86.8)
6. Fasten seat belt	17 (7.6)	207 (92.4)
7. Boarding and alighting the passengers only in bus stations	157 (70.1)	67 (29.9)
8. Pay attentions to the events happening around	165 (73.7)	59 (26.3)
9. Park the bus properly and in the designated parking zone	191 (85.3)	33 (14.7)
10. Driving in special marked lanes for buses	139 (62.1)	85 (37.9)
11. Driving within the speed limit	116 (51.8)	108 (48.2)
12. Pay attentions to pedestrians while driving	209 (93.3)	15 (6.7)
13. Avoid riding and disembarking of passengers outside the station	161 (71.9)	63 (28.1)
14. Keeping bus doors completely closed while driving	171 (76.3)	53 (7.23)
15. Wait for all passengers to take their seats before driving off	14 (6.7)	209 (93.3)
16. Avoid immediate movement after dropping off the passengers	54 (24.1)	169 (75.4)
17. Mobile phone use while driving	80 (35.7)	144 (64.3)
18. Talking to passengers while driving	36 (16.1)	188 (83.9)
19. Argument with passengers while driving	19 (8.5)	205 (91.5)
20. Boarding and alighting of passenger while waiting for the traffic light to change	60 (26.8)	164 (73.2)

Multiple linear regression analysis (enter method) was used to investigate factors influencing the incidence of unsafe behaviors. Based on the results of ANOVA, independent t-test and Pearson’s correlation between the variables of the study and the percentage of unsafe behaviors, age, working hours per day, income, emotional cycle, intellectual cycle and physical cycles of biorhythm as well as the first, second, third, fourth and fifth dimensions of the NEO personality questionnaire (neuroticism, extroversion, openness, agreeableness and conscientiousness) were eligible to enter the linear regression models (P<0.25). The results showed that the variables of emotional and intellectual biorhythm cycles would remain in the model. Based on this analysis, the emotional biorhythm cycle is likely to be the most influential factor on the incidence of unsafe behavior ( Table 7).

**Table 7:** Regression model indicating factors with influence on unsafe behaviors in the study subjects.

Variable	Unstandardized coefficients		Standardized coefficients (Beta)	t	p-value
	B	Std. Error			
Income	-0.001	0.002	-0.043	-0.30	0.76
Working hours per day	-0.045	0.408	-0.015	-0.11	0.91
Age	0.077	0.106	0.063	0.72	0.47
Neuroticism	-0.025	0.179	-0.014	-0.14	0.88
Extraversion	-0.082	0.283	-0.048	-0.291	0.77
Openness	-0.449	0.286	-0.215	-1.57	0.11
Agreeableness	0.301	0.304	0.159	0.98	0.32
Conscientiousness	-0.280	0.299	-0.163	-0.93	0.35
Intellectual cycle	6.731	3.324	0.175	2.02	0.045
Emotional cycle	8.910	3.318	0.232	2.06	0.008
Physical cycle	1.760	3.367	0.047	0.523	0.60

## Discussion

This study investigated the effect of biological rhythms and personality traits on the incidence of unsafe behaviors among bus drivers. The findings showed that the most influential factors on the percentage of unsafe behaviors were emotional and intellectual biorhythm cycles.

The mean percentage of unsafe behaviors observed among drivers was 54.8±11.91% which is relatively high, considering the nature of the driving job. Hence, it appears that special considerations are required for decreasing unsafe behaviors to curb accident rates. The percentage of unsafe behaviors in this study was approximately similar to the results of Adl et al.'s study (52.5%) and was higher compared to the previous studies conducted by Mohammadfam (42.2%) and Damyar (42.7%) on bus drivers. ^24-26^ The most observed unsafe behavior among the studied bus drivers was related to “not making sure that every passenger was occupying a sit or have a proper backrest before moving”. However, in Damyar et al.'s study, the most observed unsafe behavior was “parallel parking”^25^ but in the studies of Mohammadfam et al. and Mahmoudi et al., the most common unsafe behaviors among drivers were “driver’s talking while driving” and “smoking”, respectively.^12,26^ Due to the diversity of previously studied unsafe behaviors, similar results were unable to compare the values of unsafe behavior. In this study, there was a significant inverse correlation between unsafe behaviors and the mean scores of personality traits for all dimensions except neuroticism. In other words, with reduction in the personality trait score, unsafe behaviors also increased. This finding is consistent with the study of Mahmoudi and Haghshenas among the staff of car manufacturing companies, construction projects and drivers.^1,12^ In most of the analytical accident models, unsafe behaviors were introduced as one of the most important factors inducing accidents.^27^ Evans et al. also stated that personality traits can have an effect on driving and the rate of accidents.^28^ Therefore, the relatively high rate of unsafe behaviors obtained in this study could be attributed to personality traits' score. In this study, a negative correlation was found between unsafe behavior and extraversion scores of personality traits. This finding is in accordance with the studies of Haghshenas et al. and Hashemiyan et al.^1,9^ Moreover, a negative correlation was found between unsafe behavior and the conscientiousness scores of personality traits. One explanation is related to the fact that people with high scores on indicators of conscientiousness have more control over their desires and are able to control their impulses and actions. Therefore, they are expected to have safer driving.^9^ Moreover, the dimensions of agreeableness and openness also had a significant negative correlation with drivers' unsafe behaviors; this is in accordance with some other studies.^9,12^ It appears that individuals with higher agreeableness mean scores are basically philanthropists and are eager to help others and the people with these characteristics; therefore, they are expected to have a safer practice. Regarding the dimension of openness, psychologists believe that people with higher score in this dimension, are healthier and a driver with this characteristic may drive safer. ^9^ Finally, for the dimension of neuroticism, a significant positive correlation was found with drivers' unsafe behavior. This finding is consistent with the studies of Haghshenas et al. and Mahmoudi et al.^12,15^ This is because neurotic persons are accustomed to having negative feelings such as excitement, stress, anger, guilt and frustration and therefore, they may not sufficiently focus while driving. ^12^ The results of this study showed that there is a significant relationship between the percentages of drivers’ unsafe behaviors with the biorhythm cycle critical days. This finding is in line with previous studies.^29,32^ There is also inconsistency with some other studies.^33,34^ For instance, according to Reinhold's findings, only 2.2% of the events were likely to occur on normal days while 97.8% had a potential to be seen on the critical days. ^32^ Bordbar also indicated that biorhythm cycles and critical days had been considered as effective factors on the severity of accidents. ^35^ The days during which a cycle passes from the positive to the negative phase or vice versa are known as "critical days". Such days are known as an unstable and turbulent period in which the ability of individuals to respond to the current situation is not desirable. ^36^ As a result, the conditions are probably ready for the occurrence of accidents. ^37^ The regression modeling results also indicated that intellectual and emotional biorhythm cycles are related to the percentage of unsafe behaviors which occurred among the studied drivers. During the critical days of these cycles, the drivers had significantly committed more unsafe behaviors. These findings are consistent with the results of the Begholi’s study among power company employees in which 85% of accidents had occurred when the emotional and intellectual cycles were in critical condition.^38^ The results of this study are in accordance with the results of Latman’s study on motor vehicle drivers among which the accidents occurred more in the critical days of the intellectual and emotional cycles. ^29^ The results of Sharma and Singh's study illustrated that physical, emotional and intellectual cycles had a significant impact on the occurrence of accidents.^32^ Unlike the results of this study, the study of Mohammadfam^17^ and Zakerian^37^ indicated that the physical cycles in the automotive industry are related to the incidents and there is a significant relationship between emotional cycle and the occurrence of accidents. The reason may be due to the nature of driving activity that is mostly cognitive compared to the manufacturing industry which is mostly physical.

**Limitations of the study **

As a limitation, the cross-sectional nature of the pre-sent study does not allow actual causative conclusions to be made. In this study, self-reported measures were used which may cause some problems like deception, denial or recall. Additionally, there were difficulties for the research group to observe the bus drivers’ unsafe behaviors at the time of overcrowding which can be considered as the main limitation of this study. The re-sults of the study could be more conclusive if objective measures and more appropriate tools including video observation of driver behaviors would be included in future studies.

## Conclusion

The findings of the present study showed that the bus drivers’ emotional and intellectual biorhythm cycles are related to the incidence of unsafe behaviors. As a result, during the critical days, the occurrence of unsafe behaviors is more likely to happen. Therefore, taking measures including the selection of low-risk traffic routes in the critical days and considering the personality traits at the time of hiring individuals can be useful in reducing the rate of unsafe behaviors and accidents.
